# Supramolecular Gel as the Template for Catalysis, Inorganic Superstructure, and Pharmaceutical Crystallization

**DOI:** 10.3390/ijms20030781

**Published:** 2019-02-12

**Authors:** Arnab Dawn

**Affiliations:** James Winkle College of Pharmacy, University of Cincinnati, 231 Albert Sabin Way, Cincinnati, OH 45267-0514, USA; dawnab@ucmail.uc.edu

**Keywords:** supramolecular gels, template, catalysis, selectivity, transcription, polymorph screening

## Abstract

A supramolecular gel is a fascinating combination of flexibility and orderliness. While the supramolecular nature of crosslinking contributes towards the adaptivity and the reversibility of the system, orderliness at the molecular level amplifies the functional output and induces extraordinary selectivity into the system. Therefore, use of supramolecular gels as the soft template is an emerging area of research, which includes but not limited to catalysis of a chemical or a photochemical process, transcription of gel property to a substrate, or even controlling the nucleation of drug molecules. This review aims to highlight the template effect of supramolecular gels in the above-mentioned areas relevant to novel fundamental chemistry, technology, and healthcare.

## 1. Introduction

The journey of the fascinating chemistry supramolecular gels crossed several landmarks in the last few decades in terms of synthesis, design, characterization, and applications [1,2,3,4,5,6,7,8,9]. The very first generation of gel chemistry was focused on the synthesis of new gelator molecules, which was serendipitous in most of the cases. In the mid-90s, a limited number of groups had made the first attempts to design low molecular weight gelators [10,11,12,13,14,15]. At that stage, the gel research emphasized on new gel systems, their thermal stability and versatility in terms of gelling solvents. Then the concept of functionalization of gelator molecules towards functional application came into the picture, and supramolecular gels have been promoted as the smart materials. During this evolution of the supramolecular gel chemistry design or programming of the gelator molecules towards a specific function became the motivation for the researchers [16,17]. In this context, a significant improvement in establishing a structure-function correlation has been seen in the past two decades [18,19,20,21,22]. The outcome is simply astonishing. Now one can integrate multiple functions in a single supramolecular gel to develop complex multiresponsive systems [23,24,25,26,27]. The much-awaited complexity truly revolutionizes the fate of supramolecular gel chemistry and promotes this chemistry to facilitate and stimulate several other fields of chemistry. This review article aims to provide a quick summary of some of the most interesting developments of supramolecular gels, which demonstrate promise in manipulating this chemistry in various domains including organic, inorganic, photochemical and pharmaceutical, among many others.

An amazing combination of flexibility and structural orderliness are at the focal point of supramolecular gel chemistry. A supramolecular gel is a hierarchical build-up of individual molecules in a supramolecular manner, holding a huge number of solvent molecules. The common supramolecular connections include hydrogen-bonding, π-π stacking, metal-ligand interactions. Such primary interactions are responsible for the one-dimensional arrangement of the gelator molecules. However, the formation of a one-dimensional architecture (fibers in most of the cases) is not enough to hold the solvent molecules. Therefore, fiber entanglement to form a three-dimensional network structure is necessary for gel formation. Van der Waals interactions are the most common form of driving forces responsible for such higher order assembly which is more irregular in nature. Because of the specific nature of interactions, the primary one-dimensional molecular assembly is highly ordered. On the other hand, the higher order entanglement induces softness and adaptivity in the system. The highly organized nature of the supramolecular gels was frequently exploited for arranging functional motifs for maximizing the functional output. The functional amplification in a self-assembled state over the disintegrated molecular state has been the basis for several gel phase applications. Classical examples include developing light-harvesting systems [28,29,30,31,32,33,34] and use in molecular recognition events [35,36,37,38,39]. In most of these cases, formation of the gel was the final step of the event.

In more recent year’s researchers have improvised the use of supramolecular gels for extended operation where the formation of gels only constitutes the primary step of the process. More specifically the self-assembled molecular arrays are being used as the scaffolds for performing chemical or physical processes. Interestingly, both the substrate and the solvent have their own merits in the confined gel state. The nanostructured entangled one-dimensional assembly represents a large surface area of the substrate in direct contact with the solvent. Therefore, a substrate with an active functional group ordered in a supramolecular gel phase is capable of initiating a task where the availability of an active site with a large surface area is the key. Catalysis is an area where supramolecular gel phase materials demonstrated a huge prospect [40,41]. Transition metals are an integral part of most of the novel catalytic systems used for organic synthesis. Metallogels are the special class of supramolecular gels where the formation of primary assembly relies on the Metal-ligand coordination bond. There are several successful attempts to immobilize transition metals in a gel environment [42,43,44,45,46]. Some of those were indeed tested successfully for the catalytic process. There are certain advantages of supramolecular gel phase catalysis: (i) highly ordered catalytic site; (ii) large surface area owing to the well-defined nanostructured architecture; (iii) conformation or directional orientation of the catalytic site; (iv) slow diffusion of reactants in immobilized solvent phase. Apart from metallogels pure organogels were also utilized for the catalysis but in limited numbers. A portion of this review will discuss some interesting examples of catalytic activities of the supramolecular gel phase materials.

Apart from the conventional well-defined catalytic activity, a supramolecular gel environment is capable of facilitating various processes where the active components might or might not be the part of the gelator molecules. Supramolecular gels can act as the reaction vessels for performing photophysical and photochemical processes [47]. Among the several challenges involved in a photoinduced process, one critical issue is the product selectivity. Pre-orientation of the photoactive groups in the supramolecular gel environment prior to the photoreaction is the key difference with the isotropic solution phase process. It is postulated that such a pre-organization can’t be altered during the short life-time of the excited state species. The principles of supramolecular photochemistry [48] crucially rely on this fact. Apart from the highly ordered one-dimensional arrangement conformational restriction of the chromophores in semiflexible gel environment is another factor which contributes to the observed high degree of selectivity (or reverse selectivity) compared to the solution phase process. On the other hand, the presence of solvent molecules as an integral part of the gel ensures the restricted but desired molecular motion of the chromophore to undergo the reaction. Here, we will discuss a few very interesting works dealing with stereochemical and chiral control of photochemical processes in a supramolecular gel environment.

So far, we have discussed mainly the involvement of the active substrates within the gel for undergoing different processes. In those cases, the role of solvents is more passive. There are cases where even the solvent molecules immobilized during the gel formation can act as the active site for a chemical process. Polycondensation of immobilized silanol in the gel phase can offer a versatile route for creating silica superstructure of different shapes [49]. Other inorganic species templated by supramolecular gels include metal and metal-oxide nanostructures. We will discuss how the chemical structure and chirality of the gelator molecule influence the transcribed inorganic superstructures.

Finally, we will introduce a very recent development of the template effect of supramolecular gel phase materials as the active epitaxial surface for the crystallization of drug molecules [50]. The prerequisite of gel phase crystallization is that the gelation process occurs before the subsequent crystallization of a dissolved substrate. Gel-grown crystals can exhibit improved physical characteristics because of the suppression of convection currents and sedimentation afforded by the viscous gel environment. In many instances the gel is considered to act as an inert matrix within which crystal growth occurs, however, in specific cases, the gel structure is also capable of influencing both polymorphic form and crystal habit. In addition, the reversibility of the supramolecular gels in phase transition allows crystal recovery and re-use of the gel matrix.

The aim of the present review is not to provide an exhaustive literature survey. Instead, this is an effort to bring together available developments of template effects of supramolecular gel phase materials. Special emphasize has been given to the variety and versatility of the physical or chemical processes that are successfully doable in supramolecular gel template. Care has been taken to cover the works spanning from very first effort to very recent developments. As there is not any prior report of covering such a wide area, the author is hopeful that the present effort will encourage the researchers to exploit the supramolecular gel scaffold for even more complex processes applicable to technology generation or health care, to maintain the relevance of this field towards next generation of science. 

## 2. Supramolecular Gels for Catalyzing Chemical Processes

In a catalytic chemical transformation, a catalyst which can reduce the activation energy of the chemical event is involved. Metal complexes are particularly common for designing catalysts. They have been applied to catalyze a variety of chemical events including the efficient formation or functionalization of carbon-carbon [51], carbon-hydrogen [52], carbon-nitrogen [53] and amide bonds [54] among others. Therefore, embedding catalytic sites in terms of a metal center or a specific functional group in the gelator molecule is the most logical way of designing supramolecular gel-based catalytic systems. However, the challenge is to maintain the gelation property in presence of those active functional groups.

**Figure 1:**
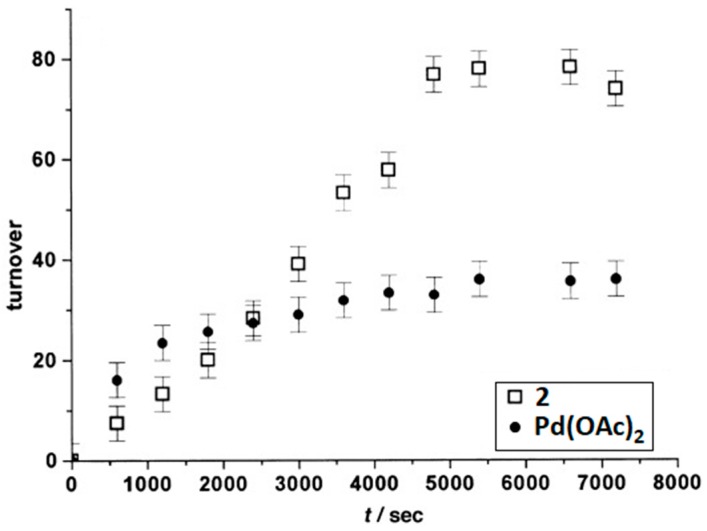
The catalytic turnovers versus time of gel produced from **2**, and Pd(OAc)_2_. Reproduced with permission from Reference [55].

**Chart 1:**
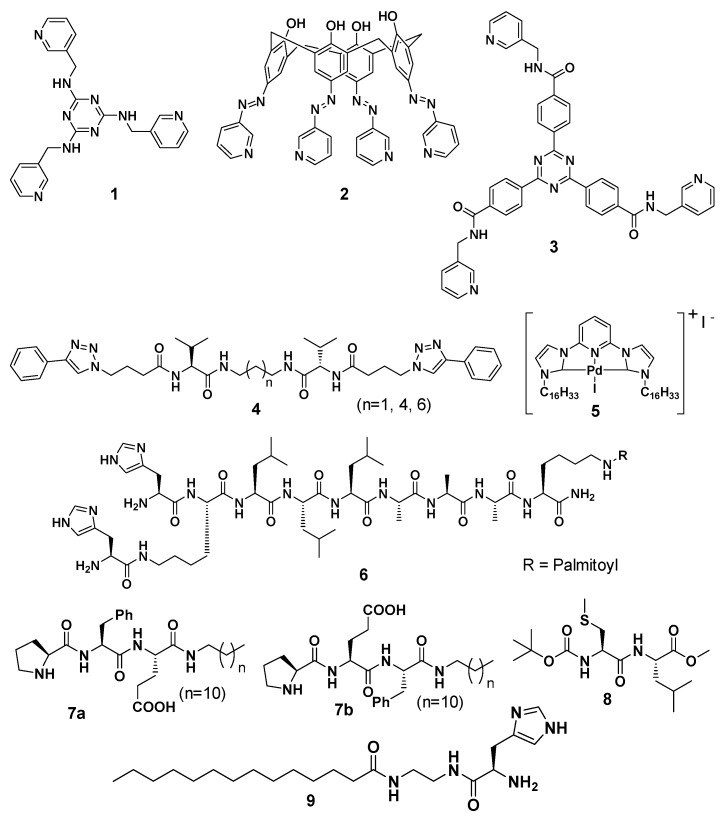
Key compounds used for developing supramolecular gel-based chemical catalytic systems.

Xu et al. reported the first example of catalytic metallogel containing Pd(II), which catalyzed the oxidative reaction of benzyl alcohol to benzaldehyde [55]. A crosslinked three-dimensional structure in DMSO was achieved by the formation of a metal-ligand coordination bond between the pyridine-based ligands **1**–**2** and Pd(OAc)_2_ (Chart 1). The catalytic turnover of the metallogel of **1** was found to be more than twice than that of the corresponding xerogel and 1.5 times higher than that of Pd(OAc)_2_. Whereas the catalytic turnover of the metallogel of **2** is twice than that of Pd(OAc)_2_, three times that of the corresponding xerogel, and four times than that of similar systems reported (Figure 1). Higher catalytic turnovers of the metallogels were related to the superior stability of the catalysts under the reaction conditions. However, the lower catalytic activity of xerogels compared with that of their corresponding gels and Pd(II) complexes was attributed to the lack of diffusion of the reaction substrates to the metal sites, and relatively smaller surface area of the insoluble coordination polymers in this heterogeneous catalytic reaction.

**Figure 2:**
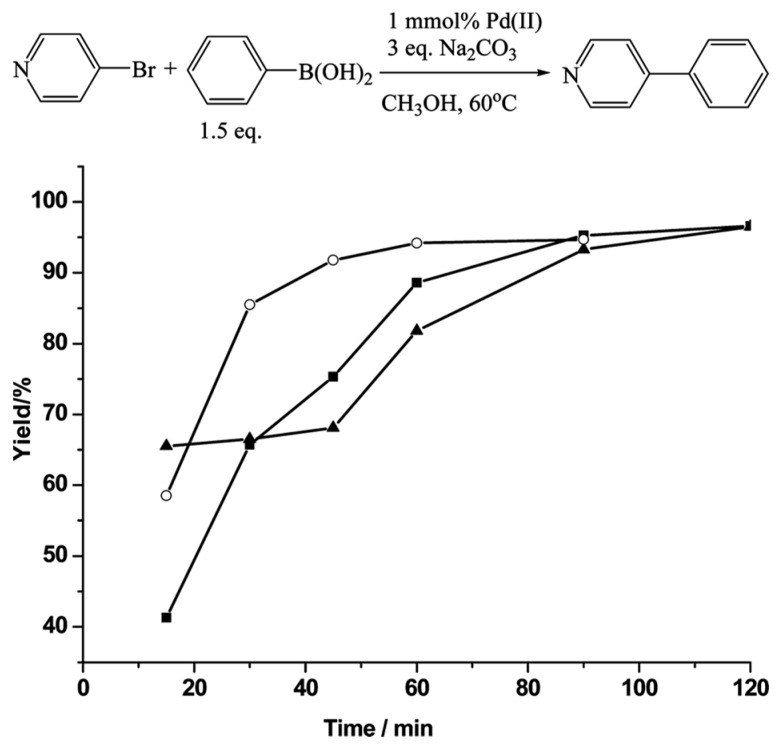
Suzuki-Miyaura coupling of 4-bromopyridine and phenylboronic acid (catalyst: circles, 1:1 gel; triangles, 1:2 gel; squares, 1:4 gel). Reproduced with permission from Reference [56].

Zhang and Su et al. reported a series of metallogels in methanol–chloroform mixtures prepared from pyridine-based tripodal ligand **3** with Pd(II) complex, which functioned as catalysts for the Suzuki-Miyaura C–C cross-coupling between 4-bromopyridine and phenylboronic acid in high yield [56]. The morphologies of the metallogels vary with metal/ligand ratio ranging from spheres to fibers. The fibrous networked gel showed higher catalytic activity compared to the spherical gel. Also, a detailed study on the catalytic activities of metallogels with different Pd/**3** ratios showed that the reaction catalyzed by the 1:1 gel took place faster than those catalyzed by the 1:4 and 1:2 gels (Figure 2). This gel-phase catalyst was also tested for the cross-coupling of other aryl halides with phenylboronic acids. It was found that the use of electron-rich aryl bromides such as iodobenzene or bromoacetophenone gave the biaryls in nearly quantitative yields, while the reaction of electron-deficient aryl bromide such as bromobenzene or 4-bromoanisole with phenylboronic acid was finished in 15 min, resulting in a relatively modest yield (28% or 26%). Interestingly, in the reaction of iodobenzene and phenylboronic acid, 1:1 xerogel maintained its catalytic activity even after at least five uses, showing better recyclable behavior than the 1:1 gel. This is just the opposite to what Xu et al. received in their catalytic process with ligand **1** and **2**, where gel appeared to be a better catalyst compared to the xerogel. In their subsequent work Zhang et al. immobilized magnetic nanoparticles (Fe_3_O_4_) in the metallogel system prepared from the same ligand **3** in presence of Pd(II) salt [57]. This system could catalyze Suzuki-Miyaura C–C coupling reactions with similar efficiency to that without the magnetic nanoparticle system. However, the gain in this strategy was that the Pd(II) xerogel could be magnetically isolated and recycled with a permanent magnet. The approach combined both the characteristics of nanofiber catalyst and magnetic nanoparticles, which offered a new solution for recycled nanoscale coordination catalysts. The gel catalyst was also employed as a catalyst in the Heckarylation of alkenes with aryl halides with excellent yields. In a more environmentally-friendly approach, very recently, Smith et al. demonstrated catalytic activity in Suzuki-Miyaura cross-coupling reaction using a hybrid hydrogel based on 1,3:2,4-dibenzylidene sorbitol modified with acyl hydrazides combined with agarose, which was capable of reducing and uptaking ‘waste’ Pd from aqueous mixtures [58].

Escuder et al. reported L-Valine-derived gelators functionalized with triazole units (**4**). The gelators upon coordinated with copper were capable of catalyzing the Huisgen 1,3-dipolar cycloaddition of alkynes and azides [59]. The obtained metallogelators exhibited a high copper loading capacity and can be regarded as efficient heterogeneous catalysts. It is interesting to note that in this study the gel formation was not dependent on the specific presence of copper. This might allow the loading of other metals having an affinity to triazole ligands without disturbing the gelation. The formation of the metallogels in water or water/alcohol mixtures also allows the reaction to be undertaken under biological conditions. Biodegradability and biocompatibility associated with amino acid derived compounds make such candidates capable as catalytic systems for bioconjugation click reactions. The catalytic activity of silver nanoparticles formed in silver coordinated metallogel systems has also been exploited by researchers in a model reduction reaction of nitrophenol [60,61].

**Scheme 1:**
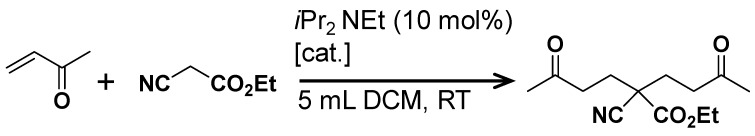
Double Michael addition.

Unlike coordination metallogels, organometallic gelators are more challenging to design and synthesis and therefore were rarely applied in catalysis. In a unique representation, Tu and Dotz et al. reported a palladium pincer bis(carbene) complex **5** which acted as an efficient organometallic gelator for a variety of protic and aprotic organic solvents in concentrations as low as 0.2 wt% [62]. π-π stacking of the heteroarene moieties, van der Waals interactions between the alkyl chains, and metal-metal interactions were the responsible factors behind the gel formation. The gel prepared from pincer complexes exhibited high catalytic efficiency in the double Michael addition of α-cyanoacetate to methyl vinyl ketone (Scheme 1). The gel phase a the crucial role in exhibiting catalytic activity as evidenced by the poor performance of the same pincer complex in a non-gel state.

**Figure 3:**
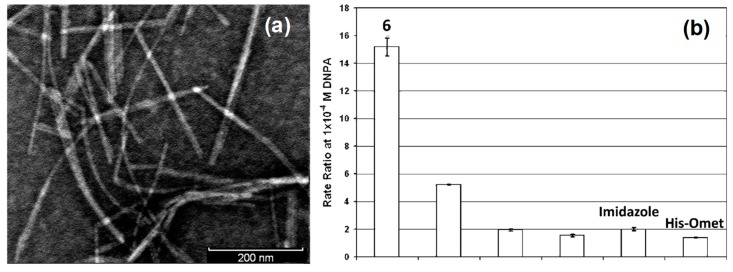
(**a**) Negatively stained TEM micrographs of a gel prepared from **6**; (**b**) observed rate increase in DNPA hydrolysis as the level of molecular organization in the catalytic particles is varied. Reproduced with permission from Reference [63].

Apart from metallogels peptide-based gels became attractive candidates for exploring catalytic activity, because of their potential to mimic enzymes. In an exciting work by Stupp et al. an amphiphile (**6**) based gel system has shown to catalyze the hydrolysis of a model compound 2,4-dinitrophenyl acetate (DNPA) [63]. Transmission electron microscopy (TEM) micrographs of the gel at pH 7.4 revealed the formation of high aspect-ratio nanostructures with the lengths ranging from hundreds of nanometers to several micrometers (Figure 3). The rate of hydrolysis in the presence of 1 × 10^−4^ M DNPA at pH 7.4 was found to be considerably higher than the other systems having spherical aggregates. This result was ascribed to the high orderliness of the active sites in the nanofibrous assembly structure. In another interesting work, Escuder et al. reported a series of short peptides bearing l-proline at their N terminus, self-assembled into high aspect ratio aggregates and formed hydrogels [64]. The aggregates produced from **7a** and **7b** were able to catalyze the aldol reaction, whereas non-aggregated analogues were catalytically inactive. Amino acid sequence, mode of organization and accessibility of the substrates to the reaction site, were found to be the key factors associated with the catalytic activity. Finally, in a biomimetic approach, the catalytic ability of these systems was successfully tested for self-condensation of α-oxy-aldehydes and phenylalkyl aldehydes. A terminally protected, hydrophobic dipeptide **8** reported by Moretto et al. was able to immobilize fullerene (C_60_) or multiwalled carbon nanotubes (MWCNTs) in the organogel by co-assembling with them [65]. The dry ternary material produced from hybrid organogel of **8** with C_60_-MWCNT was successfully tested as a catalyst for (i) the reduction reaction of water-soluble azo compounds mediated by NaBH_4_ and UV-light; and (ii) the NaBH_4_-mediated reduction of benzoic acid to benzyl alcohol. The authors correlated the self-assembly property of **8** with its tendency to occur in a single crystal structure of a six-fold screw axis. Finally, in one of the most recent examples, Banerjee et al. synthesized a histidine-based amphiphile **9** which formed hydrogels in the presence of Fe^3+^. While both, the amphiphile and the metal ion induced hydrogels demonstrated catalytic activity of p-nitrophenyl esters hydrolysis for the acetyl, n-butyl and n-octyl esters, metallo-hydrogel exhibited a higher catalytic efficiency [66].

As revealed by the above reports the key factors playing in gel phase catalysis are: (i) availability of the active sites embedded in highly ordered nanostructures with large surface area; (ii) controlled diffusion of substrates in gel environment; (iii) easy inclusion of metals in the forms of metallogels. With the number of possible ligand systems, one could imagine the options of designing metallogels are numerous. Further, with the introduction of peptide-based gelator and water as the solvent, this chemistry can potentially be extended towards more complex function and enzyme mimetics.

## 3. Supramolecular Gels for Catalyzing Photochemical Processes

Unlike in a chemical event, supramolecular gels were rarely exploited in photochemical events. There are several issues which make this task challenging. Desired orientation of the photoactive functionalities, optical transparency of the gel, absorption from the undesired functional groups, low yield, are some of the primary hurdles. Product isolation and analysis after the photoreactions are other challenges to overcome. On the positive side, highly arranged chromophores in a gel environment might offer an enhanced product selectivity over the solution state process. Here, the word catalysis refers to the superiority in performance in the context of the selectivity.

**Scheme 2:**
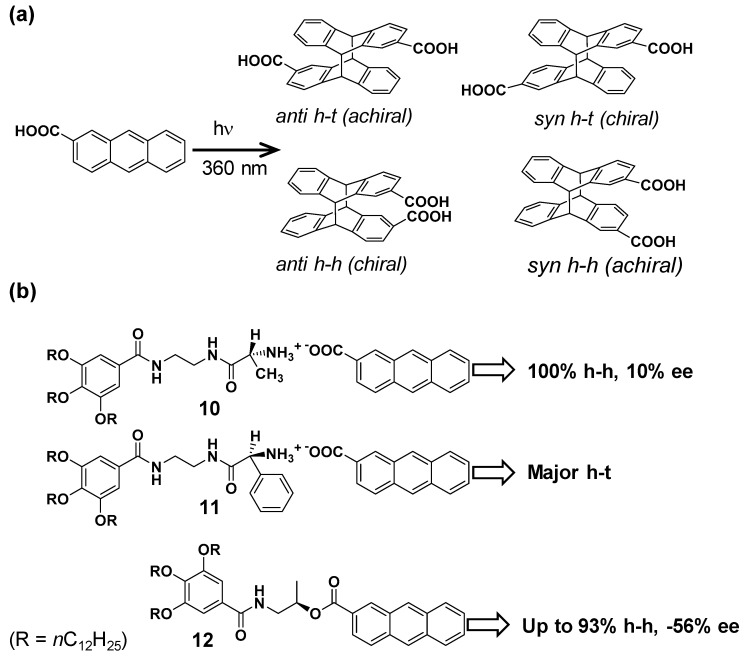
(**a**) Product distribution in a photodimerization reaction of 2-anthracnecarboxylic acid, (**b**) stereo- and enantioselectivities received using different organogel systems.

This author, with Shinkai and coworkers, reported the first example of gel phase photodimerization reaction involving an unsymmetrically substituted anthracene derivative [67]. A binary organogelator **10** has been synthesized where 2-anthracenecarboxylic acid (2Ac) was attached noncovalently with a gelator component containing the gallic acid backbone coupled with D-alanine by means of electrostatic and hydrogen-bonding interactions. While in the isotropic state always head-to-tail (h-t) are the major photodimers, photodimerization of 2Ac in cyclohexane gel showed the unprecedented stereochemical selectivity, resulting in only head-to-head (h-h) photocyclodimers exclusively together with significant enantiomeric excess induced by the chiral counterpart of the gelator (Scheme 2). As the consequence of the process, a photoinduced gel-to-sol phase transition took place. The π-π stacking together with the intermolecular hydrogen bonding via two amide linkages cooperatively constructed the one-dimensional arrangement of the gelator molecules, which was facilitated by the nonpolar solvent environment. The preferential preorientation prior to the photoreaction played a crucial role in selectivity. In the subsequent work using the binary gelator **11** where the alanine moiety was replaced by a bulky phenylglycine group, the stereoselectivity could be reversed towards h-t photodimers [68]. Here the steric effect of the gelator changed the preorientation. Finally, to improve the chiral factor, the organogelator **12** containing 2-anthracnecarboxylic acid attached covalently with a gelator counterpart consists of 3,4,5-*tris*(n-dodecan-1-yloxy)-benzoic acid via a chiral amino alcohol linkage has been synthesized [69]. Photodimers isolated after the photoreaction of the gel samples executed different degrees of stereoselectivities. In the gel state, the formation of h-h photodimes was always favored over h-t photodimers. Enantiomeric excess (ee) value of the major h-h photodimes reached as high as -56% in case of the gels with enantiomeric glycidyl methyl ethers. However, it was found that the solvent chirality was outweighed by the intrinsic chirality of the gelator molecules. Moreover, the stereochemistry of the photodimers could be tuned simply by the solvent selection This was the first successful demonstration of supramolecular photochirogenesis performed in the gel state.

**Chart 2:**
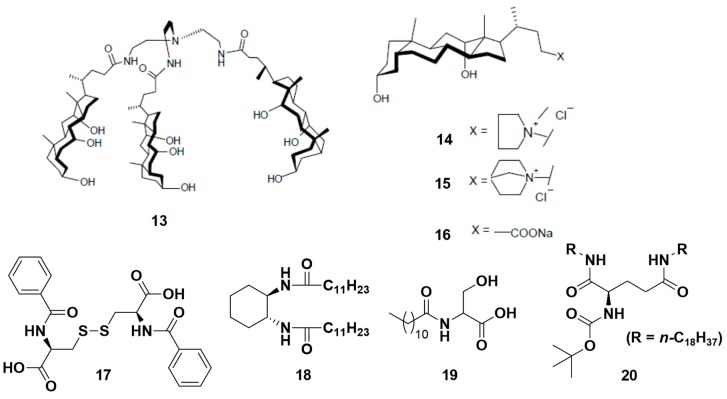
Various compounds used for the supramolecular gel-based photochemical process.

Earlier Maitra et al. reported photodimerization of acenaphthylene using bile acid derived gelators **13**–**16** (Chart 2). The ratio of anti to syn photodimers was found to be greater in the gel bound state than in solution [70]. Selectivity of the photochemical process was found to be related to the rigidity of the gels. However, the selectivity was lower compared to the reactions reported in clay or zeolites. Later, Díaz Díaz et al. demonstrated use of low molecular weight hydrogel and organogel templates using the compound **16**–**19** for the Riboflavin tetraacetate (RFT)-catalyzed aerobic photooxidation of 1-(4-methoxyphenyl)ethanol under LED blue visible light irradiation [71]. Photooxidation rates in hydrogel media were on average 10–20-fold lower than those in stirred solutions, but only 1–3-fold lower under non-stirred conditions. Using the approach where the catalyst was physically entrapped with and the substrate in the gel environment, the conversions were between 55% and 100% within 120 min. Surprisingly, in acetonitrile gel with bisamide gelator 15 the reaction gives 100% conversion only within 1 h without the need of thiourea and stirring. In contrast to the reaction carried out in acetonitrile solution, the gel environment also prevented deactivation of the catalyst. Interestingly, the gelators could be recycled without affecting their gelation ability and reaction conversion. Moreover, kinetics could be fine-tuned according to the characteristics of the gel media.

More recently, D´ıaz D´ıaz et al. demonstrated that useful C–C bond-forming photoredox catalysis could be performed in air using gel networks as reaction media to give similar results as could be obtained under inert atmosphere conditions [72]. Functionalization of aryl halides using rhodamine-6G (Rh-6G) as photocatalyst involves the generation of the ground-state Rh-6G·– radical anion in the presence of an electron donor under green-light irradiation and the excited Rh-6G·–* radical anion upon blue-light irradiation. Instability of Rh-6G radical anions in the presence of oxygen requires a strictly oxygen-free environment. In the above study, such limitation was overcome by including a small amount of gelator **20** in the reaction mixture, which transformed the solution into a viscoelastic gel medium. Fibrillar gel networks confined the reactants and inhibited oxygen diffusion, allowing air-sensitive catalytic activity under ambient conditions. The combination of enhanced viscosity and added interfaces in supramolecular gel media appeared to be the key factor in facilitating the reactions under aerobic conditions.

As evident from only a limited number of reports, the use of supramolecular gel media for the photochemical process is still an underexplored area. The real challenge is to determine a structure-function correlation to fine-tune a photochemical event, which is quite different from a chemical process. However, by thoughtful design, even a simple steric interference can change the course of a photochemical process as evident in one of the works presented above. Chirality is another factor which can be treated in a unique way in a supramolecular gel environment via induction or transcription in a supramolecular manner. Finally, highly organized nanostructures together with the restricted solvent environment offering controlled diffusion can have a huge potential of controlling the kinetics of a photochemical event and determining the product selectivity.

## 4. Supramolecular Gels as the Surface for Fabricating Inorganic Superstructures

**Chart 3:**
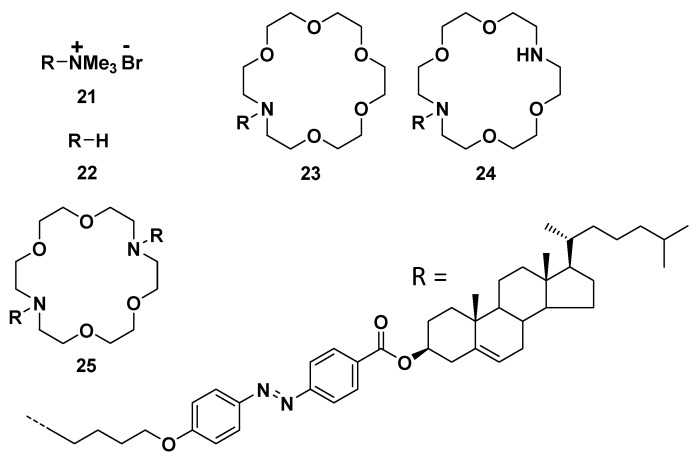
Cholesterol-based organogelators used for creating silica superstructures.

**Figure 4:**
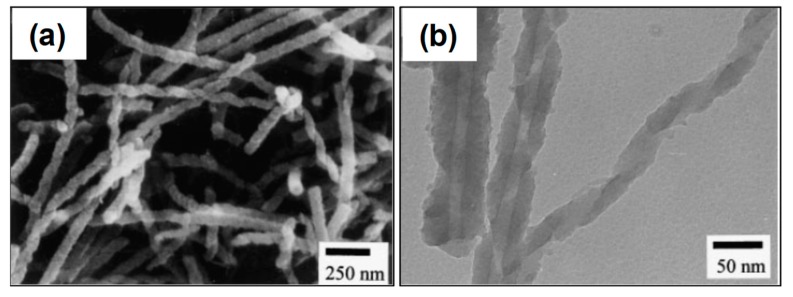
(**a**) SEM and (**b**) TEM images of silica structures prepared using the mixture of **21** and **22** as a template for **21**/(**21** + **22**) = 10 mol%. Reproduced with permission from Reference [75].

Inorganic materials frequently lack in the well-defined superstructure, especially with defined morphology. To overcome this issue, the use of organic templates to control the inorganic superstructures has been a hot topic in past decades [49]. One of the several advantages in organic templating is the easy removal of the template from the desired structured inorganic material. Tunable nanostructures based on structure-function correlation made the supramolecular gel a promising candidate to template inorganic superstructures. Shinkai and co-workers pioneered in this area and reported the first successful example of tubular silica structure using an acetic acid gel of **21** in the presence of tetraethoxysilane (TEOS) and water. Subsequent drying and calcination steps resulted in hollow, tubular silica with inner diameters comparable to those of the original gel fibers, and free of organic template [73]. Presence of the cationic charge in **21** facilitating the adsorption of anionic silica under acidic condition was found to be indispensable for the transcription event. Interestingly, using mixed organogels of **21** and **22** (nonionic gelator) hollow, helical silica could only be obtained at certain ratios of the two organogelators (Chart 3) [74]. A high abundance of cationic charges in the gel fibers resulted in random adsorption of anionic silica precursors or particles, whereas a moderate concentration resulted in excellent transcription property. A very low concentration, on the other hand, did not result in any transcription. Very interestingly, fibrous silica with a right-handed ‘‘helical’’ structure was created only when **21**/(**21** + **22**) = 5–15 mol% (Figure 4). The presence of extra cationic charges also appeared to disrupt the transcription when present in high concentrations [75].

**Figure 5:**
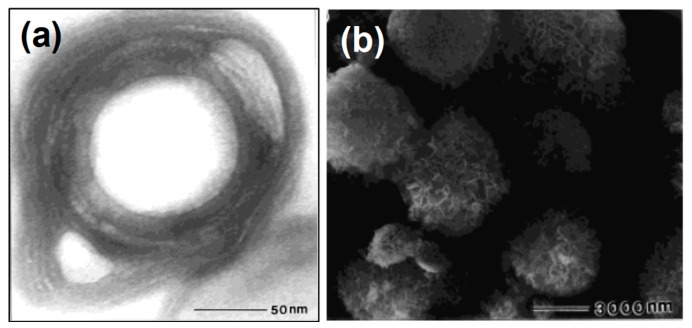
(**a**) TEM micrograph of the silica obtained from the gel with **25** in acetic acid after sectioning, (**b**) SEM micrograph of the silica obtained from the gel with **25**/Pd(NO_3_)_2_ in aniline. Reproduced with permission from Reference [78].

Multi-layered ‘rolled-paper-like’ silica has been prepared using the aza[18]crown-6-functionalized gelator **23** [76]. The organogel of the diaza-analogue **24**, on the other hand, consisted of fully formed tubular structures [77]. In absence of any positive charges, the driving force for the transcriptions with **23** and **24** was the cationic charges arising from protonation of the azacrown moieties. Multiwalled silica spheres have been transcribed using the organogel prepared from of the diazacrown-appended cholesterol based gelators **25** (Figure 5a) [78]. Interestingly, the organogel of **25** in presence of Pd(NO_3_)_2_ in aniline resulted in a fluffy, globular silica structure (Figure 5b). Electron probe microanalysis (EPMA) studies clearly evidenced the uniform distribution of Pd throughout the silica matrix. Such process might be useful in creating metal catalytic sites on the silica support.

**Chart 4:**
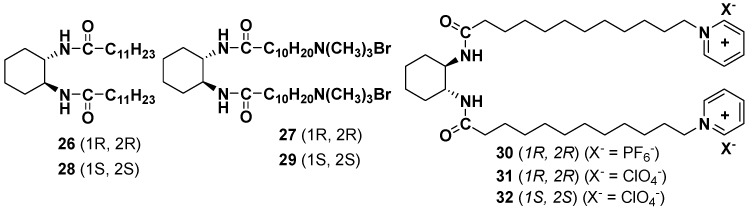
Diaminocyclohexane-based organogelators used for creating inorganic superstructures.

**Figure 6:**
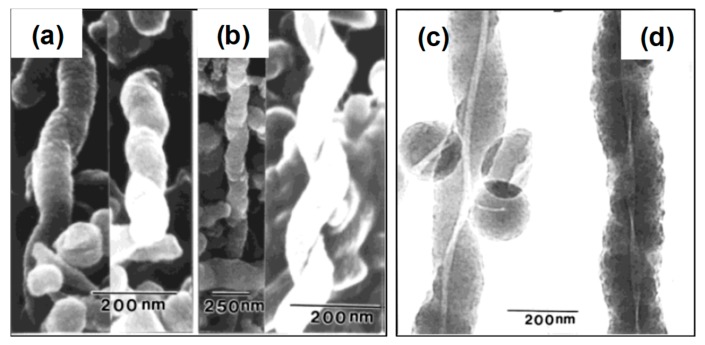
SEM and TEM micrographs of the silica obtained by sol-gel transcription in (**a**,**c**) left-handed **26** + **27** (1:1 wt%), and (**b**,**d**) right-handed **28** + **29** (1:1 wt%) organogels. Reproduced with permission from Reference [79].

**Figure 7:**
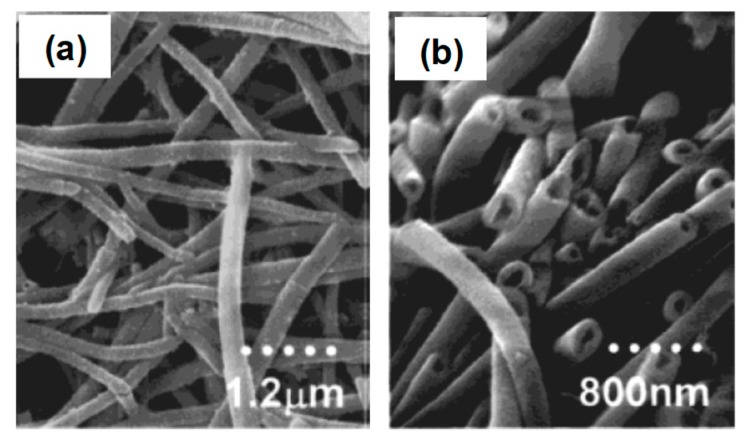
SEM images of the samples prepared under basic conditions: (**a**) dried sample from **30** (**b**) calcined sample. Reproduced with permission from Reference [80].

**Figure 8:**
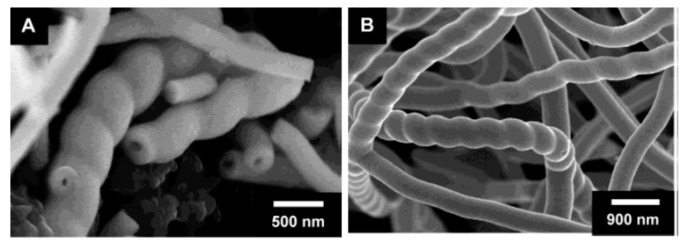
SEM images of tantalum oxide fibers obtained from (**A**) (*R*,*R*)-enantiomer **31** and (**B**) (*S*,*S*)-enantiomer **32**. Reproduced with permission from Reference [81].

Apart from cholesterol-based gelator diaminocyclohexane-based organogelators first reported by Hanabusa et al. offer well defined chiral nanostructures. Right-handed and left-handed helical silica superstructure have been created by Shinkai and Hanabusa et al. by using mixtures of eninantiomeric compounds **26** (neutral) with **27** (positively charged), and **28** (neutral) with **29** (positively charged) (Chart 4 and Figure 6) [79]. Interestingly, the transcription was found to be successful only when the presence of a charged cationic compound was between 30–85%. A very high abundance of charged species inhibited the gel formation whereas a minimum amount of charged species was necessary for transcription as evident from the previous discussion. Sol-gel polymerization of Ti[OCH(CH_3_)_2_]_4_. using the gel template made of **30** resulted hollow titania tubes with outer diameters of 150–600 nm and inner diameters of 50–300 nm, where the latter corresponded to the diameters of the gel template fibers (Figure 7) [80]. Fibrous gel structure and electrostatic interaction between anionic titania species and cationic gelator under basic conditions were the key factors in achieving inorganic superstructure. Using similar approach, the sol-gel polymerization of metal (Ti, Ta, V) alkoxides using organogelators **31** and **32** as templates afforded tubular helical materials of transition-metal (Ti, Ta, V) oxides (Figure 8) with handedness defined by the gelator chirality [81].

**Chart 5:**
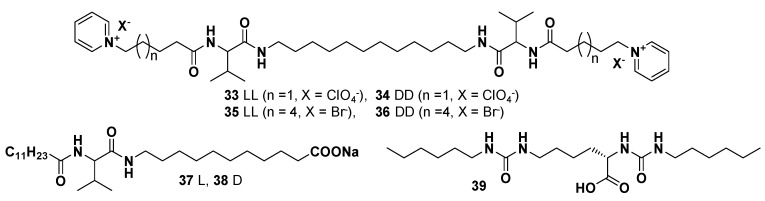
Amphiphiles used for templating inorganic superstructures.

**Figure 9:**
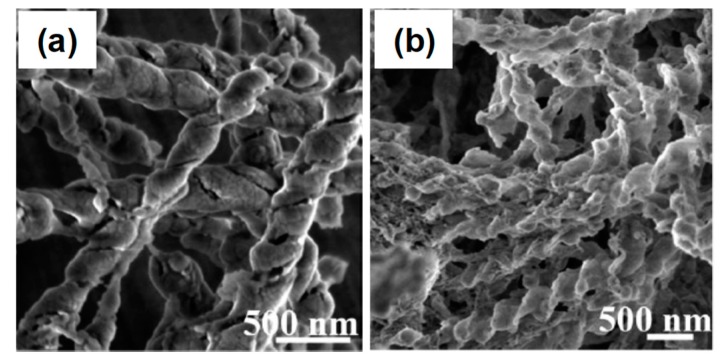
FE-SEM images of the (**a**) right- and (**b**) left-handed helical ZrO_2_ nanotubes and double coiled nanoribbons after being calcined at 700 °C for 3.0 h. Reproduced with permission from Reference [83].

Single-handed helical CdS nanotubes were prepared by Li and Yang et al. via sol-gel transcription approach using the organogelators **33** and **34** (Chart 5). The handedness of the nanotubes was controlled by the handedness of the organic self-assemblies [82]. Electrostatic interaction between Cd^2+^ and perchloride was proposed to drive the adsorption of Cd^2+^ on the surfaces of the chiral organic templates. Single-handed helical ZrO_2_ nanotubes with crystalline walls were prepared using the same gelator pair (Figure 9) [83]. The calcined nanotubes had mixed monoclinic and tetragonal structures. Adsorption of ZrO_2_ by pyridinium rings of the gelator molecules through electrostatic interactions and chirality transfer by amide groups to the inner surfaces of the ZrO_2_ nanotubes through a C=O/Zr^4+^ interaction were the key factors behind this transcription event. Later, Single-handed twisted tubular Pt nanoribbons were prepared through sol-gel transcription, using self-assemblies of amphiphiles **35** and **36** [84]. Nanoribbons were constructed from randomly organized nanoparticles and short nanowires with diameters of 1.5–3.0 nm. More recently, single-handed twisted 4,4-biphenylene-bridged polybissilsesquioxane tubular nanoribbons and single-layered nanoribbons were reported via supramolecular templating approach using enantiomeric gelators **37** and **38** [85]. A handedness inversion took place during the preparation process. Carbonization and removal of silica resulted in carbonaceous tubular nanoribbons and single-layered nanoribbons. The CD spectra indicated that the twisted tubular nanoribbons exhibited optical activity, while the twisted single-layered nanoribbons did not. Here, chirality is transferred from the gelator self-assemblies to the inner surfaces of the 4,4’-biphenylene-bridged polybissilsesquioxane tubular nanoribbons and subsequently to those of the carbonaceous tubular nanoribbons.

**Figure 10:**
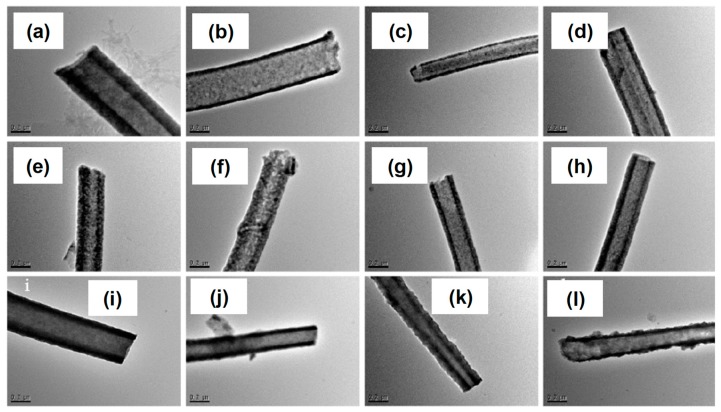
TEM images of metal oxide/TiO_2_ hybrid nanotubes (**a**–**d**: Zr, **e**–**h**: Nb, and **i**–**l**: Si). Ti:Zr = 9:1 (**a**); 8:2 (**b**); 7:3 (**c**) and 6:4 (**d**); Ti:Nb = 9:1 (**e**), 8:2 (**g**) and 6:4 (**h**); Ti:Si = 9:1 (**i**), 8:2 (**j**), 7:3 (**k**) and 6:4 (**l**). Scale bars are 0.2 µm. Reproduced from Reference [86].

In a very recent work by Suzuki and Hanabusa et al., fabrication of TiO_2_ nanotubes and hybridization with other metal oxides was achieved via the sol-gel method using an l-lysine organogelator **39** as the template [86]. The crystalline structures of the TiO_2_ nanotubes changed from anatase into rutile with increasing calcination temperatures. The hybrid nanotubes of TiO_2_ with Ta_2_O_5_, ZrO_2_, Nb_2_O_5_, and SiO_2_ were fabricated by the sol-gel polymerization in ethanol gels containing two raw materials (Figure 10). As a result of the hybridization, the surface areas increased, and the crystalline phase transition temperature from anatase to rutile became high. In another recent report, negatively-charged self-assembling peptide amphiphile was used as a template to nucleate calcium phosphate mineralization. Acidic peptide molecules were shown to induce the formation of hydroxyapatite like calcium phosphate mineralization [87].

**Chart 6:**
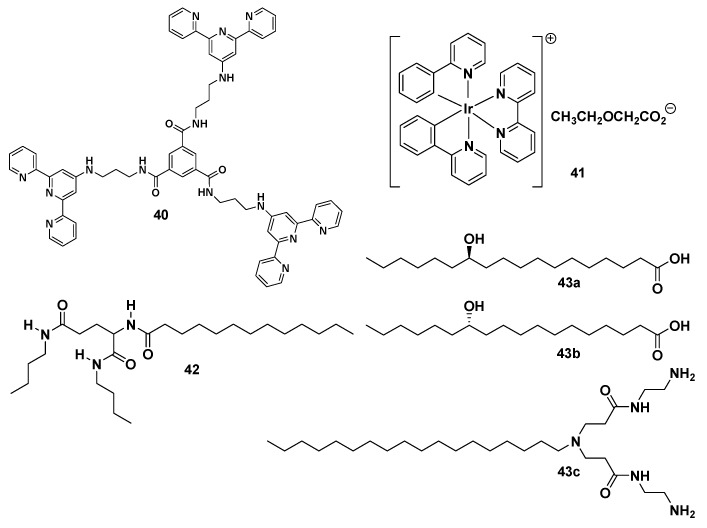
Various compounds used in self-assembly for creating inorganic superstructure.

Apart from organic gelators, organic-inorganic hybrid gel systems were also used for templating inorganic nanostructures. Boland et al. demonstrated the growth of single-crystal halide salt nanowires with diameters ∼130–200 nm., facilitated by a self-assembled supramolecular gel made of ligand **40** and EuCl_3_ (Chart 6). The inclusion of the halide salts in the supramolecular gel led to the formation of seed crystals and diffusion-driven base growth of nanowires [88]. A hydrogel based on the Ir(III) complex **41** reported by Godbert et al., consisted of tetragonal columnar strands formed by Ir(III) cations, which were self-assembled in a columnar oblique system [89]. Upon complete dehydration of the gel phases, the obtained xerogel thin films maintained identical supramolecular architectures. The gels were deposited through spin-coating onto quartz-substrates and IrO_2_ thin films were obtained by calcination of the spin-coated xerogels. The IrO_2_ samples obtained using the most concentrated gels showed an inner ordered nanostructure.

In another interesting example, a combination of an amino acid ionic liquid tetramethylammonium glycinate and gelator **42** were used as a templating agent for the fabrication of mesoporous alumina with diverse morphologies via a solvothermal synthetic method [90]. While ionic liquid only and gelator only systems resulted in irregular sheets and hierarchical clusters, a distinct cactus rod-like morphology with interlaced lateral plates was constructed using the combination of two. It was proposed that the ionic liquid components promoted the self-assembly of **42** and interacted with its linear structure via strong intermolecular hydrogen bonds, which resulted in the formation of a bilayer arrangement. Then the AlOOH nanocrystals could be adsorbed to form a hybrid with the ionic liquid, and induced axial growth along the bilayer.

**Figure 11:**
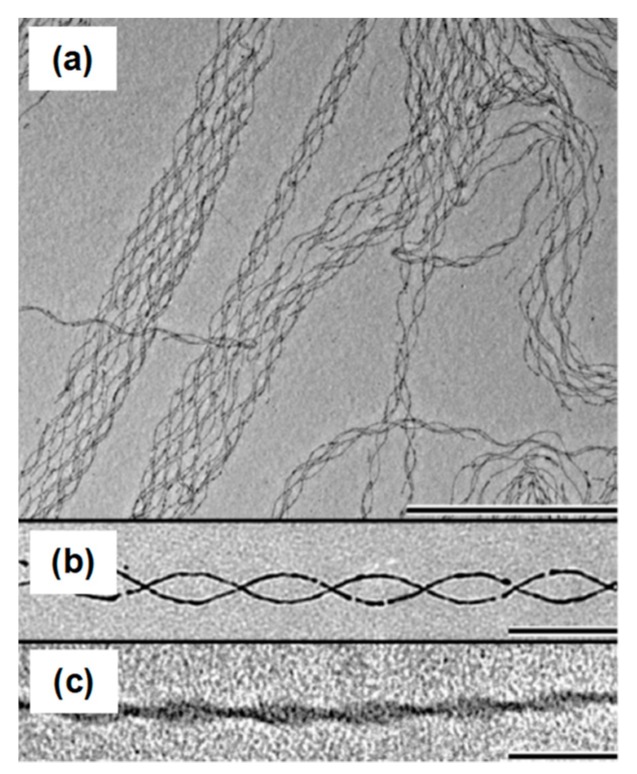
(**a**,**b**) High- and low-magnification TEM images of double-helical Au NWs and (**c**) a twisted nanoribbon composed of **43a** and **43c**. Scale bars: (**a**) 500 nm and (**b**,**c**) 100 nm. Reproduced with permission from Reference [91].

Finally, in an exciting example Kawai et al. succeeded in transmitting structure scaffold as well as chirality to generate double helical Au nanowires (Figure 11) [91]. The reported chiral soft-templates composed of two types of organogelators **43a**–**43c** where first two enantiomeric components served to introduce helicity into the template and the third one acted as the capping agent to control the Au shape.

Starting from solely SiO_2_ the transcription of gel structure is now extended to more varieties of inorganic materials. This is the evidence of the versatility in this approach. Another unique advantage of the gel-based template is the ease of chirality transfer. Finally, easy synthetic modifications of this class of gelators can generate an extensive library of organic soft templates with variable morphologies, which in turn can be applied to fine-tune the inorganic superstructure towards technological applications.

## 5. Supramolecular Gels as the Surface for Pharmaceutical Crystallization

The role of the gel in a crystallization process is multifaceted. Firstly, a viscoelastic gel medium slows down the convection current facilitating slow diffusion of the substrate. Secondly, one-dimensional ordering offered by gel fibers provides an active, high energy surface on which substrate nucleation can occur epitaxially in such a way that the periodicity of the gel fibre is transferred to the growing crystal. Another added advantage of supramolecular gels in crystallization is its reversibility which offers easy recovery of crystals and multiple uses of the gel matrix.

**Chart 7:**
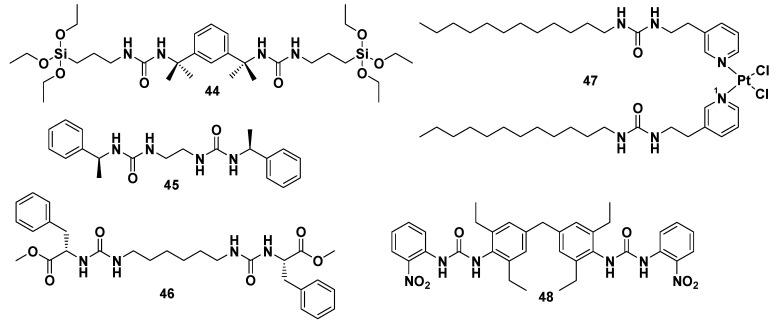
Compounds used for supramolecular gel phase crystallization.

**Figure 12:**
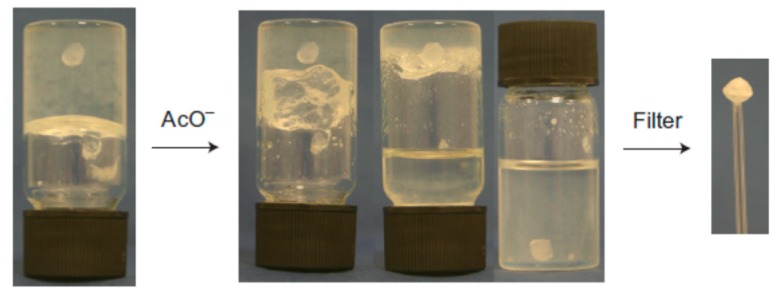
Recovery of a single crystal of CBZ form III by acetate-anion triggered gel dissolution of a 1:9 CHCl_3_: toluene gel of gelator **46**. Reproduced with permission from Reference [92].

The first supramolecular gel phase crystallization was reported by the Steed group. In this work, the growth of a range of crystals of organic compounds, including pharmaceuticals, was achieved in bis(urea) gels [92]. Clear differences in crystal habits have been seen in the gel phase when compared with the solution. In some cases, the gel-phase crystallization resulted in the formation of different polymorphs when compared to the control experiments performed in solution. Conversion of kinetically stable needle-like form II of carbamazepine (CBZ) to the thermodynamically favorable form III was found to be significantly retarded in the gel of **44** prepared in toluene (Chart 7). This observation was related to the limited convection in the gel samples, slowing down dissolution of the kinetic form. Also, the crystals grown inside the gelator network exhibited a tendency to more well-defined crystal faces and larger sizes. Recovery of the CBZ crystals grown in the gel phase was readily achieved by adding excess tetrabutylammonium acetate to destroy the gel (Figure 12). Acetone gels of compound **45** in two weeks resulted in a large single crystal of CBZ characterized as the triclinic acetone solvate. A control experiment in pure acetone did not produce any crystals. Interestingly, an unusual CBZ crystal of form I was formed using a gel of compound **46** in DMSO/water mixture. Form I is the high-temperature form and is normally produced from the melt at 170 °C. Also, dehydration of the dihydrate can lead to form I under conditions of low humidity. The formation of pure form I in the gel was a good indication that a supramolecular gel environment can have a significant effect on crystal form. Among the other compounds, aspirin was crystallized from a gel of **45** in acetonitrile while an equally concentrated solution of aspirin in acetonitrile did not crystallize under the same conditions, suggesting that the gel can be used to induce crystallization. The crystals of 2-hydroxybenzyl alcohol obtained from the gel of **46** in toluene were significantly larger, better developed and with more regular faces, compared to that in toluene solution.

**Figure 13:**
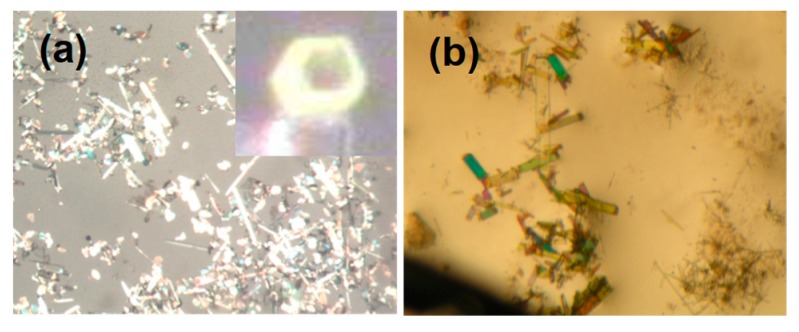
Optical microscope images (90× magnification) of the cisplatin crystals grown at the gel-sol interface using the o-xylene gel of **47**: (**a**) DMF solvate of cisplatin (Inset: magnified photo of the crystal with novel morphology), and (**b**) DMA solvate of cisplatin. Reproduced with permission from Reference [93].

Compared to the generic gelators used in the above study, in a more target-specific approach, a dug mimetic gelator can potentially influence the substrate crystallization to a greater extent owing to its seeding effect arising from structural similarity. The first study of supramolecular gel phase crystallization using a substrate mimetic approach has been reported by this author and Steed group [93]. In this study, a series of platinum-based low-molecular-weight urea-based gelators, mimicking the structure of the anticancer drug cisplatin has been synthesized. Morphological and rheological studies established that the inclusion of a longer spacer between the urea and cisplatin-mimetic regions of the gelator resulted in optimal gelation performance. Interfacial crystallization of cisplatin in a gel-sol biphasic system has been employed to address the insolubility of cisplatin in organic solvents. A new *N*,*N*-dimethylacetamide (DMA) solvate of cisplatin has been identified and a crystal habit modification of the known *N*,*N*-dimethylformamide (DMF) solvate form of cisplatin has been observed on crystallization of cisplatin in the gel of **47** prepared in xylenes (Figure 13). A control experiment with a generic gelator was also performed. While both drug mimetic and generic gels resulted in the formation of the new DMA solvate, only the cisplatin mimetic gel prepared from **47** resulted in high-quality single crystal suitable for characterization by single crystal crystallography. The structural and geometrical resemblance between **47** and cisplatin together with the ordered assembly in this gel enhanced the influence of the **47** on cisplatin crystallization. The mode of interaction between the gelator and the substrate might involve a combination of Pt-Pt stacking and amine-chloride hydrogen bonding interactions.

Using the above approach, in another recent study, the Steed group reported a bis(urea) gelator **48** designed to mimic the chemical structure of highly polymorphic drug substance ROY [94]. Crystallization of ROY from toluene gels of the gelator resulted in the formation of the metastable red form instead of the thermodynamic yellow polymorph. Under identical conditions, crystallization from generic gels, from solution and from solutions containing the designer gelator at sub-critical gelation concentration all produced the thermodynamic form. Conformational and crystal structure prediction methods showed that the templation of the red form by the drug mimetic gel resulted from conformational matching of the gelator to the ROY coupled with overgrowth of ROY onto the local periodic structure of the gel fibres.

Vitamin B_9_ gel was used by Mei et al. as the co-crystallization media, resulting in four stoichiometric vitamin C co-crystals [95]. Under highly supersaturated conditions, uniform microspheres of vitamin C-nicotinamide with different particle sizes were obtained through a spherical crystallization process.

**Figure 14:**
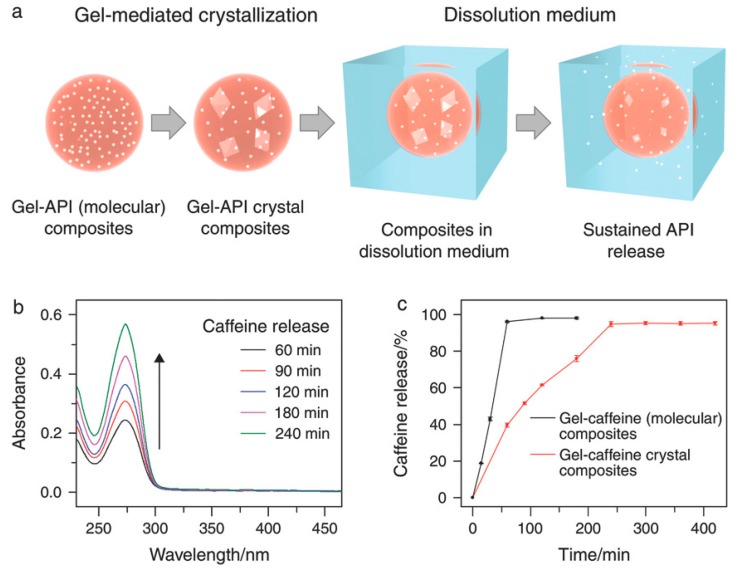
(**a**) Illustration of the process for sustained drug release from the TA–Ti(IV)–drug crystal Composites, (**b**) UV–vis absorption spectra of the caffeine released from the TA–Ti(IV)–caffeine crystal composite particles over time, (**c**) graph showing the release profiles of caffeine from the gel–caffeine composites. Reproduced with permission from Reference [96].

Caruso et al. reported a tannic acid–Ti (IV) supramolecular metallogel system which was the first metallogel system prepared from a natural organic gelator without the need of a heat–cool trigger and used as a medium for crystallization of the model drugs caffeine, carbamazepine, and piroxicam [96]. Gel-mediated crystallization resulted in crystals with different sizes, morphologies, and polymorphs when compared with those formed in solutions. Interestingly, these gel–drug crystal composites were further tested for the sustained release of model drug caffeine (Figure 14).

From the above examples, it is clear that supramolecular gel phase crystallization has all the potential to emerge as an alternative polymorph screening technique. This technique is particularly useful for a substance which is difficult to crystallize or to isolate a metastable form of a known drug with higher pharmaceutical merit. This technique also offers a fine-tuning of the crystal habit for an enhanced bioavailability or a longer shelf life.

## 6. Outlook

New generation supramolecular gel phase materials promise a lot beyond its traditional domain of serendipity. Synthetic tuneability together with molecular engineering approach and extraordinary orderliness in molecular domain made this chemistry applicable to various sectors such as technology, healthcare, pharmaceutical. As depicted here the template effect of this class of materials can equally be applied in chemical and photocatalysis, designing inorganic superstructure, and even in drug polymorph screening. Easy substrate recovery, recyclability of template materials adds even more to its multifaceted presence across the disciplines.

## References

[1] Dawn A., Shiraki T., Haraguchi S., Tamaru S.-I., Shinkai S. (2011). What kind of “soft materials” can we design from molecular gels?. Chem. Asian J..

[2] Babu S.S., Praveen V.K., Ajayaghosh A. (2014). Functional π-gelators and their applications. Chem. Rev..

[3] Jones C.D., Steed J.W. (2016). Gels with sense: Supramolecular materials that respond to heat, light and sound. Chem. Soc. Rev..

[4] Yu G., Yan X., Han C., Huang F. (2013). Characterization of supramolecular gels. Chem. Soc. Rev..

[5] Okesola B.O., Smith D.K. (2016). Applying low-molecular weight supramolecular gelators in an environmental setting-selfassembled gels as smart materials for pollutant removal. Chem. Soc. Rev..

[6] Draper E.R., Adams D.J. (2017). Low-molecular-weight gels: the state of the art. Chemistry.

[7] Liu M., Ouyang G., Niu D., Sang Y. (2018). Supramolecular gelatons: Towards the design of molecular gels. Org. Chem. Front..

[8] Draper E.R., Adams D.J. (2018). How should multicomponent supramolecular gels be characterised?. Chem. Soc. Rev..

[9] Mayr J., Saldıas C., Dıaz Dıaz D. (2018). Release of small bioactive molecules from physical gels. Chem. Soc. Rev..

[10] Murata K., Aoki M., Suzuki T., Harada T., Kawabata H., Komori T., Ohseto F., Ueda K., Shinkai S. (1994). Thermal and light control of the sol-gel phase transition in cholesterol-based organic gels. Novel helical aggregation modes as detected by circular dichroism and electron microscopic observation. J. Am. Chem. Soc..

[11] Snijder C.S., de Jong J.C., Meetsma A., van Bolhuis F., Feringa B.L. (1995). A novel low molecular weight chiral gelator for apolar organic solvents. Chem. Eur. J..

[12] Hanabusa K., Yamada M., Kimura M., Shirai H. (1996). Prominent gelation and chiral aggregation of alkylamides derived from trans-1,2-diaminocyclohexane. Angew. Chem. Int. Ed..

[13] Van Esch J.H., Schoonbeek F., de Loos M., Kooijman H., Spek A.L., Kellogg R.M., Feringa B.L. (1999). Cyclic bis-urea compounds as gelators for organic solvents. Chem. Eur. J..

[14] Shi C., Huang Z., Kilic S., Xu J., Enick R.M., Beckman E.J., Carr A.J., Melendez R.E., Hamilton A.D. (1999). The gelation of CO_2_: A sustainable route to the creation of microcellular materials. Science.

[15] Luboradzki R., Gronwald O., Ikeda M., Shinkai S., Reinhoudt D.N. (2000). An attempt to predict the gelation ability of hydrogen-bond-based gelators utilizing a glycoside library. Tetrahedron.

[16] Van Esch J.H., Feringa B.L. (2000). New functional materials based on self-assembling organogels: From serendipity towards design. Angew. Chem. Int. Ed..

[17] Dastidar P. (2008). Supramolecular gelling agents: can they be designed?. Chem. Soc. Rev..

[18] Dawn A., Kumari H. (2018). Low molecular weight supramolecular gels under shear: Rheology as the tool for elucidating structure–function correlation. Chem. Eur. J..

[19] Malliaa V.A., Weiss R.G. (2016). Correlations between thixotropic and structural properties of molecular gels with crystalline networks. Soft Matter.

[20] Dawn A., Shiraki T., Ichikawa H., Takada A., Takahashi Y., Tsuchiya Y., Lien L.T.N., Shinkai S. (2012). Stereochemistry-dependent, mechanoresponsive supramolecular host assemblies for fullerenes: A guest-induced enhancement of thixotropy. J. Am. Chem. Soc..

[21] Meyer A.R., Bender C.R., dos Santos D.M., Ziembowicz F.I., Frizzo C.P., Villetti M.A., Reichert J.M., Zanatta N., Bonacorso H.G., Martins M.A.P. (2018). Effect of slight structural changes on the gelation properties of N-phenylstearamide supramolecular gels. Soft Matter.

[22] Fang W., Sun Z., Tu T. (2013). Novel supramolecular thixotropic metallohydrogels consisting of rare metal–organic nanoparticles: synthesis, characterization, and mechanism of aggregation. J. Phys. Chem. C.

[23] Sun Z., Huang Q., He T., Li Z., Zhang Y., Yi L. (2014). Multistimuli-responsive supramolecular gels: Design rationale, recent advances, and perspectives. ChemPhysChem.

[24] Fan K., Wang X., Yin Z., Jia C., Zhang B., Zhou L., Song J. (2018). Novel two-component gels with multi-stimuli response: the gel–sol phase transition and color changes. J. Mater. Chem. C.

[25] Ren Y., Xie S., Grape E.S., Inge A.K., Ramstrom O. (2018). Multistimuli-responsive enaminitrile molecular switches displaying H+-induced aggregate emission, metal ion-induced turn-on fluorescence, and organogelation properties. J. Am. Chem. Soc..

[26] Borre E., Stumb J.-F., Bellemin-Laponnaz S., Mauro M. (2016). Light-powered self-healable metallosupramolecular soft actuators. Angew. Chem. Int. Ed..

[27] Wang T., Yu X., Li Y., Ren J., Zhen X. (2017). Robust, self-healing, and multistimuli-responsive supergelator for the visual recognition and separation of short-chain cycloalkanes and alkanes. ACS Appl. Mater. Interfaces.

[28] Ghosh S., Praveen V.K., Ajayaghosh A. (2016). The Chemistry and Applications of π-Gels. Annu. Rev. Mater. Res..

[29] Diaz D.D., Saldias C. (2018). Photon upconversion in supramolecular gels and synthetic application. Curr. Org. Chem..

[30] Guerzo A.D., Olive A.G.L., Reichwagen J., Hopf H., Desvergne J.-P. (2005). Energy transfer in self-assembled [n]-acene fibers involving ≥100 donors per acceptor. J. Am. Chem. Soc..

[31] Sugiyasu K., Fujita N., Shinkai S. (2004). Visible-light-harvesting organogel composed of cholesterol-based perylene derivatives. Angew. Chem. Int. Ed..

[32] Praveen V.K., Ranjith C., Armaroli N. (2014). White-light-emitting supramolecular gels. Angew. Chem. Int. Ed..

[33] Felip-León C., Díaz-Oltra S., Galindo F., Miravet J.F. (2016). Chameleonic, light harvesting photonic gels based on orthogonal molecular fibrillization. Chem. Mater..

[34] Duan P., Yanai N., Nagatomi H., Kimizuka N. (2015). Photon upconversion in supramolecular gel matrixes: Spontaneous accumulation of light-harvesting donor–acceptor arrays in nanofibers and acquired air stability. J. Am. Chem. Soc..

[35] Gambhir D., Kumar S., Dey G., Krishnan V., Koner R.R. (2018). Preferential intermolecular interactions lead to chiral recognition: enantioselective gel formation and collapse. Chem. Commun..

[36] Mukhopadhyay P., Iwashita Y., Shirakawa M., Kawano S.-I., Fujita N., Shinkai S. (2006). Spontaneous colorimetric sensing of the positional isomers of dihydroxynaphthalene in a 1D organogel matrix. Angew. Chem. Int. Ed..

[37] Chen J.-F., Liu X., Ma J.-F., Han B.-B., Ding J.-D., Lin Q., Yao H., Zhang Y.-M., Wei T.-B. (2017). A pillar[5]arene-based multiple-stimuli responsive metal–organic gel was constructed for facile removal of mercury ions. Soft Matter.

[38] Zhang L., Jin Q., Liu M. (2016). Enantioselective recognition by chiral supramolecular gels. Chem. Asian J..

[39] Lin Q., Lu T.T., Zhu X., Wei T.B., Li H., Zhang Y.M. (2016). Rationally introduce multi-competitive binding interactions in supramolecular gels: a simple and efficient approach to develop multi-analyte sensor array. Chem. Sci..

[40] Escuder B., Rodríguez-Llansola F., Miravet J.F. (2010). Supramolecular gels as active media for organic reactions and catalysis. New J. Chem..

[41] Fang W., Zhang Y., Wu J., Liu C., Zhu H., Tu T. (2018). Recent advances in supramolecular gels and catalysis. Chem. Asian J..

[42] Tam A.Y.-Y., Yam V.W.-W. (2013). Recent advances in metallogels. Chem. Soc. Rev..

[43] Dastidar P., Ganguly S., Sarkar K. (2016). Metallogels from coordination complexes, organometallic, and coordination polymers. Chem. Asian J..

[44] Häringa M., Díaz D.D. (2016). Supramolecular metallogels with bulk self-healing properties prepared by in situ metal complexation. Chem. Commun..

[45] Winter A., Schubert U.S. (2016). Synthesis and characterization of metallo-supramolecular polymers. Chem. Soc. Rev..

[46] Jung J.H., Lee J.H., Silverman J.R., John G. (2013). Coordination polymer gels with important environmental and biological applications. Chem. Soc. Rev..

[47] Pérez-Ruiz R., Díaz D.D. (2015). Photophysical and photochemical processes in 3D self-assembled gels as confined microenvironments. Soft Matter.

[48] Yang C., Inoue Y. (2014). Supramolecular photochirogenesis. Chem. Soc. Rev..

[49] van Bommel K.J.C., Friggeri A., Shinkai S. (2003). Organic templates for the generation of inorganic materials. Angew. Chem. Int. Ed..

[50] Kumar D.K., Steed J.W. (2014). Supramolecular gel phase crystallization: orthogonal self-assembly under non-equilibrium conditions. Chem. Soc. Rev..

[51] Cherney A.H., Kadunce N.T., Reisman S.E. (2015). Enantioselective and enantiospecific transition-metal-catalyzed cross-coupling reactions of organometallic reagents to construct C–C bonds. Chem. Rev..

[52] Ping L., Chung D.S., Bouffard J., Li S.-G. (2017). Transition metal-catalyzed site- and regio-divergent C–H bond functionalization. Chem. Soc. Rev..

[53] Wang Q., Su Y., Li L., Huang H. (2016). Transition-metal catalysed C–N bond activation. Chem. Soc. Rev..

[54] Serrano E., Martin R. (2018). Forging amides through metal-catalyzed C–C coupling with isocyanates. Eur. J. Org. Chem..

[55] Xing B., Choi M.-F., Xu B. (2002). Design of coordination polymer gels as stable catalytic systems. Chem. Eur. J..

[56] Liu Y.-R., He L., Zhang J., Wang X., Su C.-Y. (2009). Evolution of spherical assemblies to fibrous networked Pd(II) metallogels from a pyridine-based tripodal ligand and their catalytic property. Chem. Mater..

[57] Liao Y., He L., Huang J., Zhang J., Zhuang L., Shen H., Su C.-Y. (2010). Magnetite nanoparticle-supported coordination polymer nanofibers: Synthesis and catalytic application in Suzuki-Miyaura coupling. ACS Appl. Mater. Interfaces.

[58] Slavık P., Kurka D.W., Smith D.K. (2018). Palladium-scavenging self-assembled hybrid hydrogels–reusable highly-active green catalysts for Suzuki–Miyaura cross-coupling reactions. Chem. Sci..

[59] Araujo M., Diaz-Oltra S., Escuder B. (2016). Triazolyl-based molecular gels as ligands for autocatalytic ‘Click’ reactions. Chem. Eur. J..

[60] Lee J.H., Kang S., Lee J.Y., Jung J.H. (2012). A tetrazole-based metallogel induced with Ag^+^ ion and its silver nanoparticle in catalysis. Soft Matter.

[61] Paul M., Sarkar K., Dastidar P. (2015). Metallogels derived from silver coordination polymers of C3-symmetric tris(pyridylamide) tripodal ligands: synthesis of Ag nanoparticles and catalysis. Chem. Eur. J..

[62] Tu T., Assenmacher W., Peterlik H., Weisbarth R., Nieger M., Dotz K.H. (2007). An air-stable organometallic low-molecular-mass gelator: Synthesis, aggregation, and catalytic application of a palladium pincer complex. Angew. Chem. Int. Ed..

[63] Guler M.O., Stupp S.I. (2007). A self-assembled nanofiber catalyst for ester hydrolysis. J. Am. Chem. Soc..

[64] Tena-Solsona M., Nanda J., Díaz-Oltra S., Chotera A., Ashkenasy G., Escuder B. (2016). Emergent catalytic behavior of self-assembled low molecular weight peptide-based aggregates and hydrogels. Chem. Eur. J..

[65] Mazzier D., Carraro F., Crisma M., Rancan M., Toniolo C., Moretto A. (2016). A terminally protected dipeptide: From crystal structure and self-assembly, through co-assembly with carbon-based materials, to a ternary catalyst for reduction chemistry in water. Soft Matter.

[66] Gayen K., Basu K., Bairagi D., Castelletto V., Hamley I.W., Banerjee A. (2018). Amino-acid-based metallo-hydrogel that acts like an esterase. ACS Appl. Bio Mater..

[67] Dawn A., Fujita N., Haraguchi S., Sada K., Shinkai S. (2009). An organogel system can control the stereochemical course of anthracene photodimerization. Chem. Commun..

[68] Dawn A., Fujita N., Haraguchi S., Sada K., Tamaru S.-I., Shinkai S. (2009). Studies on a new class of organogelator containing 2-anthracenecarboxylic acid: Influence of gelator and solvent on stereochemistry of the photodimers. Org. Biomol. Chem..

[69] Dawn A., Shiraki T., Haraguchi S., Sato H., Sada K., Shinkai S. (2010). Transcription of chirality in the organogel systems dictates the enantiodifferentiating photodimerization of substituted anthracene. Chem. Eur. J..

[70] Bhat S., Maitra U. (2007). Hydrogels as reaction vessels: Acenaphthylene dimerization in hydrogels derived from bile acid analogues. Molecules.

[71] Bachl J., Hohenleutner A., Dhar B.B., Cativiela C., Maitra U., Ko¨nig B., Dıaz D.D. (2013). Organophotocatalysis in nanostructured soft gel materials as tunable reaction vessels: comparison with homogeneous and micellar solutions. J. Mater. Chem. A.

[72] Häring M., Abramov A., Okumura K., Ghosh I., König B., Yanai N., Kimizuka N., Díaz D.D. (2018). Air-sensitive photoredox catalysis performed under aerobic conditions in gel networks. J. Org. Chem..

[73] Ono Y., Nakashima K., Sano M., Kanekiyo Y., Inoue K., Hojo J., Shinkai S. (1998). Organic gels are useful as a template for the preparation of hollow fiber silica. Chem. Commun..

[74] Ono Y., Nakashima K., Sano M., Hojo J., Shinkai S. (1999). Template effect of cholesterol-based organogels on sol-gel polymerization creates novel silica with a helical structure. Chem. Lett..

[75] Ono Y., Nakashima K., Sano M., Hojo J., Shinkai S. (2001). Organogels are useful as a template for the preparation of novel helical silica fibers. J. Mater. Chem..

[76] Jung J.H., Ono Y., Shinkai S. (1999). Novel preparation method for multi-layered, tubular silica using an azacrown-appended cholesterol as template and metaldeposition into the interlayer space. J. Chem. Soc. Perkin Trans. 2.

[77] Jung J.H., Ono Y., Shinkai S. (2000). Novel silica structures which are prepared by transcription of various superstructures formed in organogels. Langmuir.

[78] Jung J.H., Ono Y., Sakurai K., Sano M., Shinkai S. (2000). Novel vesicular aggregates of crown-appended cholesterol derivatives which act as gelators of organic solvents and as templates for silica transcription. J. Am. Chem. Soc..

[79] Jung J.H., Ono Y., Hanabusa K., Shinkai S. (2000). Creation of both right-handed and left-handed silica structures by sol-gel transcription of organogel fibers comprised of chiral diaminocyclohexane derivatives. J. Am. Chem. Soc..

[80] Kobayashi S., Hanabusa K., Hamasaki N., Kimura M., Shirai H., Shinkai S. (2000). Preparation of TiO_2_ hollow-fibers using supramolecular assemblies. Chem. Mater..

[81] Kobayashi S., Hamasaki N., Suzuki M., Kimura M., Shirai H., Hanabusa K. (2002). Preparation of helical transition-metal oxide tubes using organogelators as structure-directing agents. J. Am. Chem. Soc..

[82] Zhang C., Huo H., Li Y., Li B., Yang Y. (2013). Preparation of helical CdS nanotubes using a sol–gel transcription approach. Mater. Lett..

[83] Huo H., Wang S., Lin S., Li Y., Li B., Yang Y. (2014). Chiral zirconia nanotubes prepared through a sol–gel transcription approach. J. Mater. Chem. A.

[84] Cai H., Wang C., Li B., Li Y., Yang Y. (2014). Preparation and characterization of single-handed twisted platinum tubular nanoribbons. Mater. Lett..

[85] Liu D., Li B., Guo Y., Li Y., Yang Y. (2015). Inner surface chirality of single-handed twisted carbonaceous tubular nanoribbons. Chirality.

[86] Suzuki M., Tanaka K., Kato Y., Hanabusa K. (2017). Metal oxide/TiO_2_ hybrid nanotubes fabricated through the organogel Route. Gels.

[87] Eren E.D., Tansik G., Tekinay A.B., Guler M.O. (2018). Mineralized peptide nanofiber gels for enhanced osteogenic differentiation. ChemNanoMat.

[88] Daly R., Kotova O., Boese M., Gunnlaugsson T., Boland J.J. (2013). Chemical nano-gardens: growth of salt nanowires from supramolecular self-assembly gels. Acs Nano.

[89] Scarpelli F., Ionescu A., Aiello I., La Deda M., Crispini A., Ghedini M., Brunelli E., Sesti S., Godbert N. (2017). High order in a self-assembled iridium(III) complex gelator towards nanostructured IrO_2_ thin films. Chem. Asian J..

[90] Lei X., Liu T., Tang S. (2018). Fabrication of cactus rod-like mesoporous alumina with ionic liquid-supramolecular gelator as cotemplate. Cryst. Growth Des..

[91] Nakagawa M., Kawai T. (2018). Chirality-controlled syntheses of double-helical Au nanowires. J. Am. Chem. Soc..

[92] Foster J.A., Piepenbrock M.-O.M., Lloyd G.O., Clarke N., Howard J.A.K., Steed J.W. (2010). Anion-switchable supramolecular gels for controlling pharmaceutical crystal growth. Nat. Chem..

[93] Dawn A., Andrew K.S., Yufit D.S., Hong Y., Reddy J.P., Jones C.D., Aguilar J.A., Steed J.W. (2015). Supramolecular gel control of cisplatin crystallization: Identification of a new solvate form using a cisplatin-mimetic gelator. Cryst. Growth Des..

[94] Foster J.A., Damodaran K.K., Maurin A., Day G.M., Thompson H.P.G., Cameron G.J., Bernal J.C., Steed J.W. (2017). Pharmaceutical polymorph control in a drug-mimetic supramolecular gel. Chem. Sci..

[95] Wang J.-R., Bao J., Fan X., Dai W., Mei X. (2016). pH-Switchable vitamin B9 gels for stoichiometry controlled spherical co-crystallization. Chem. Commun..

[96] Rahim M.A., Hata Y., Björnmalm M., Ju Y., Caruso F. (2018). Supramolecular metal–phenolic gels for the crystallization of active pharmaceutical ingredients. Small.

